# Double prenylation of budding yeast Ykt6 regulates cell wall integrity and autophagy

**DOI:** 10.1016/j.jbc.2025.108384

**Published:** 2025-03-04

**Authors:** Masaki Tateishi, Kota Goto, Eiji Hishinuma, Naomi Matsukawa, Takuma Kishimoto, Kazuma Tanaka, Hisanori Horiuchi, Masayoshi Fukasawa, Ryutaro Shirakawa

**Affiliations:** 1 Department of Molecular and Cellular Biology, Institute of Development, Aging and Cancer, Tohoku University, Sendai, Japan; 2 Advanced Research Center for Innovations in Next-Generation Medicine, Tohoku University, Sendai, Japan; 3 Tohoku Medical Megabank Organization, Tohoku University, Sendai, Japan; 4 Division of Molecular Interaction, Institute for Genetic Medicine, Graduate School of Life Science, Hokkaido University, Sapporo, Japan; 5 Laboratory of Organelle Pathophysiology, Department of Integrative Life Sciences, Graduate School of Life Sciences, Tohoku University, Sendai, Japan; 6 Department of Biochemistry and Cell Biology, National Institute of Infectious Diseases, Tokyo, Japan

**Keywords:** SNARE proteins, Ykt6, protein prenyltransferase, protein isoprenylation, MALDI-TOF/TOF, membrane trafficking, glycosyltransferase, cell wall, autophagy, organelle proteome

## Abstract

Ykt6 is a conserved SNARE protein involved in multiple membrane trafficking pathways, including intra-Golgi transport and autophagic membrane fusion. We previously demonstrated that mammalian Ykt6 is uniquely modified with farnesyl and geranylgeranyl groups at two C-terminal cysteines through the sequential action of farnesyltransferase (FT) and geranylgeranyltransferase type 3 (GGT3). Although these two cysteines are strictly conserved in all eukaryotes, the evolutionary conservation of Ykt6 double prenylation remains unclear, as budding yeast appears to lack the α subunit of GGT3. In this study, we used structural predictions to identify the uncharacterized protein Ecm9 as the functional α subunit of yeast GGT3. Ecm9 forms a complex with Bet2 and transfers a geranylgeranyl group to mono-farnesylated Ykt6. MALDI-TOF/TOF mass spectrometry confirmed that budding yeast Ykt6 is doubly prenylated with farnesyl and geranylgeranyl groups in wild-type cells but not in *ecm9Δ* cells. Loss of Ecm9 resulted in fragile cell walls, likely due to mislocalization of Golgi mannosyltransferases. Furthermore, *ecm9Δ* cells exhibited impaired Ykt6 localization to organelle membranes including autophagosomes, leading to reduced autophagic activity. These findings establish that double prenylation is an evolutionarily conserved structural feature of Ykt6 and is essential for its membrane localization and function.

Protein prenylation is a major post-translational lipid modification in eukaryotes, occurring in approximately 1% of human proteins (1, 2, 3). This process involves the irreversible attachment of either a 15-carbon farnesyl group or a 20-carbon geranylgeranyl group to cysteine residues near the C-terminus of target proteins. Prenylation enables the association of target proteins with lipid bilayers and, in some cases, directly mediates protein-protein interactions (4). Examples of prenylated proteins include members of the Ras superfamily of small GTPases, the γ subunits of trimeric GTP-binding proteins, nuclear lamins, and various other proteins involved in cellular signaling.

Protein prenylation is catalyzed by protein prenyltransferases. In mammals, four types of prenyltransferases have been identified: farnesyltransferase (FT), geranylgeranyltransferase type 1 (GGT1), geranylgeranyltransferase type 2 (GGT2, also known as RabGGT), and the recently discovered geranylgeranyltransferase type 3 (GGT3) (5, 6). These enzymes function as heterodimers, each composed of an α subunit and a β subunit. FT and GGT1 share the same α subunit but differ in their β subunits. FT and GGT1 recognize a consensus sequence called the CAAX box at the C-terminus of protein substrates, where C represents cysteine, A is typically an aliphatic residue, and X determines substrate specificity. After prenylation, the terminal AAX tripeptide is proteolytically cleaved by Ras-converting enzyme 1 (RCE1), a membrane protease located in the endoplasmic reticulum (ER). The free carboxyl group of the prenylated cysteine is then methylated by isoprenylcysteine carboxyl methyltransferase (ICMT) using S-adenosylmethionine (SAM) as a methyl donor. These post-prenylation processes increase the hydrophobicity of the C-terminus of CAAX proteins. GGT2 specifically targets the Rab family of small GTPases with the help of chaperone proteins called Rab escort proteins (REPs). Rab proteins typically possess two cysteines at their C-terminus (*e.g.*, CXC, CC, or CCXX), both of which are geranylgeranylated by GGT2. The fourth enzyme, GGT3, contains a unique α subunit termed prenyltransferase α subunit repeat containing 1 (PTAR1) and shares the same β subunit with GGT2. In a previous study, we identified the SNARE protein Ykt6 as the sole protein substrate for GGT3 (6).

SNARE proteins serve as the minimal machinery required for intracellular membrane fusion (7). SNAREs contain one or two conserved helical regions, called SNARE motifs, which assemble into a parallel four-helix bundle known as the SNARE complex. This complex drives membrane fusion by bringing opposing membranes into close proximity. The central layer of the SNARE complex, known as the zero layer, consists of three glutamine (Q) residues and one arginine (R) residue. Based on the amino acid composition of the zero layer, SNAREs are classified as either Q-SNAREs or R-SNAREs. Ykt6 is the most highly conserved R-SNARE protein and is widely distributed among eukaryotic species.

Ykt6 was originally identified as a farnesylated SNARE protein involved in ER-to-Golgi transport in budding yeast (8). Subsequent studies in yeast and mammals have revealed the versatile roles of Ykt6 in multiple membrane trafficking pathways (9), including intra-Golgi transport (10, 11, 12, 13), Golgi-to-vacuole/lysosome transport (14, 15), homotypic vacuolar fusion (16), and post-Golgi trafficking (17). More recently, Ykt6 has been shown to localize to autophagosomes under starvation conditions, where it regulates their fusion with vacuoles/lysosomes (18, 19, 20, 21).

Ykt6 consists of an N-terminal globular longin domain and a C-terminal SNARE domain (22). Unlike most SNARE proteins, Ykt6 lacks a C-terminal transmembrane domain and instead contains a conserved tandem cysteine motif (C^194^C^195^AIM in mammals). Cys195 is farnesylated by FT, followed by cleavage of the AIM tripeptide and subsequent C-terminal methylation. Although early studies proposed that Cys194, adjacent to the farnesylated cysteine, undergoes reversible palmitoylation (23, 24, 25), we have demonstrated that Cys194 is irreversibly geranylgeranylated by GGT3 in human cells and rat tissues (6). Consequently, mammalian Ykt6 is doubly prenylated with farnesyl and geranylgeranyl groups. Despite these hydrophobic modifications, Ykt6 remains soluble and is predominantly localized in the cytosol. Structural studies indicate that Ykt6 adopts a closed conformation in the cytosol, with its prenyl groups sequestered in a hydrophobic groove formed between the longin and SNARE domains (26, 27). While the mechanism by which Ykt6 switches to an open conformation and associates with membranes remains incompletely understood, recent studies suggest that phosphorylation regulates its conformational states and subsequent SNARE complex formation (28, 29, 30, 31, 32).

The C-terminal tandem cysteine motif of Ykt6 is evolutionarily conserved across eukaryotes (*e.g.*, C^196^C^197^IIM in budding yeast). However, PTAR1 appears to be present only in species ranging from flies to humans and is not found in budding yeast. This suggests that double prenylation of Ykt6 might be specific to metazoans rather than a universal feature of eukaryotes. In budding yeast, previous studies have reported that Ykt6 undergoes reversible palmitoylation at Cys196 and that its conformational changes and subcellular localization are dynamically regulated by a cycle of palmitoylation and depalmitoylation (33). However, as palmitoylation of endogenous Ykt6 has not been directly demonstrated in yeast and Ykt6 was not detected in a comprehensive yeast palmitoylome analysis (34), the possibility of Cys196 geranylgeranylation remains. Since lipid modification affects the conformational changes of Ykt6, determining its lipid modification in budding yeast is essential for a unified understanding of its mechanism of action.

In this study, we used structural predictions to identify Ecm9, a previously uncharacterized protein in budding yeast, as the structural and functional homolog of human PTAR1. Mass spectrometry analysis revealed that budding yeast Ykt6 is stably modified with both farnesyl and geranylgeranyl groups. In *ecm9Δ* yeast cells, Ykt6 failed to localize to organelle membranes, leading to defects in Golgi enzyme localization and autophagy. Our findings demonstrate that double prenylation is a conserved structural feature of Ykt6 across eukaryotes and is essential for its regulatory function.

## Results

### Conserved C-terminal cysteines of Ykt6 are essential for yeast growth

**Figure 1:**
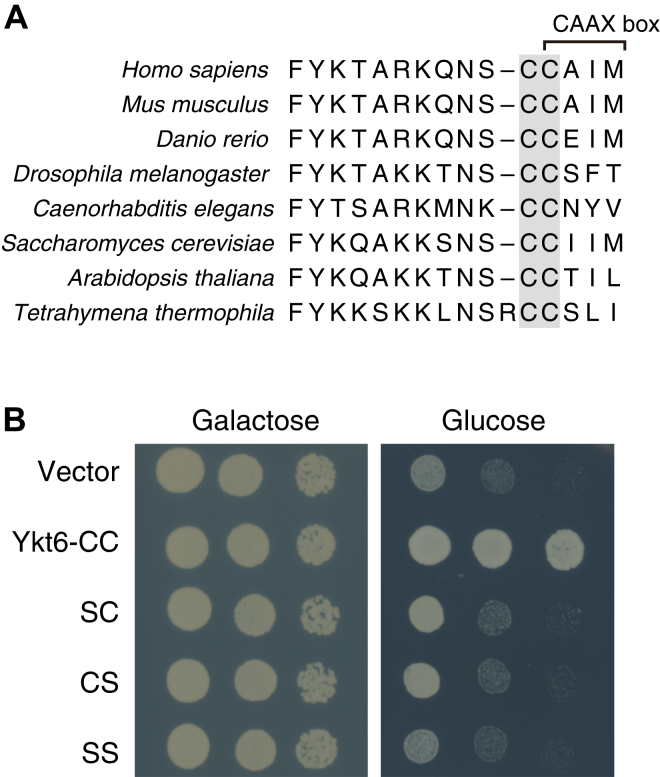
**Conserved two cysteines at the C-terminus of Ykt6 are essential for yeast growth.***A*, multiple sequence alignment of the C-terminal sequence of Ykt6 from various species. The two conserved cysteine residues are highlighted in *gray*. *B*, yeast growth assay showing the essential role of the two cysteine residues. A yeast strain with Ykt6 expression under the control of the *GAL1* promoter was transformed with either an empty vector or vectors expressing wild-type Ykt6 (CC), Ykt6^C196S^ (SC), Ykt6^C197S^ (CS), or Ykt6^C196/197S^ (SS). Serial dilutions of the cultures were spotted onto galactose or glucose agar plates to assess yeast growth.

Figure 1*A* shows a multiple sequence alignment of the C-terminal region of Ykt6 from various species, highlighting the broad conservation of two C-terminal cysteines across eukaryotes. Previous studies have shown that both cysteines are essential for yeast growth (8, 33). To confirm the functional importance of these cysteines, we used a yeast strain expressing *YKT6* under the control of the *GAL1* promoter. Since *YKT6* is an essential gene, this strain cannot grow in a glucose medium. As shown in Figure 1*B*, Ykt6 mutants with one or both cysteines replaced by serines failed to support yeast growth in the glucose medium, confirming that both cysteines are indispensable. These results suggest that the cysteine immediately preceding the CAAX box, Cys196, undergoes a critical post-translational modification in budding yeast.

### Budding yeast Ecm9 is a structural homolog of human PTAR1

**Figure 2:**
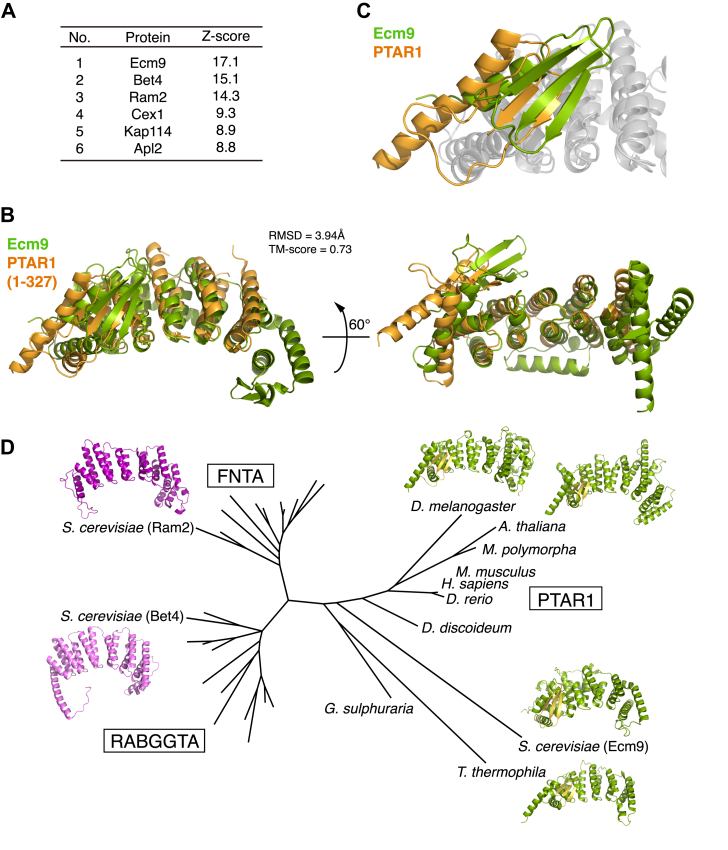
**Ecm9 is a structural homolog of PTAR1.***A*, results of a DALI search for structural homologs of PTAR1 in budding yeast. The search was performed against the AlphaFold protein structure database using the crystal structure of human PTAR1 (PDB: 6J6X) as a query. Proteins are ranked by Z-scores, which reflect structural similarity. *B*, structural alignment of human PTAR1 and yeast Ecm9. The crystal structure of PTAR1 (residues 1–327, *orange*) is aligned with the AlphaFold-predicted structure of Ecm9 (*green*). The root mean square deviation (RMSD) and template modeling (TM)-score are shown. Structures with a TM-score higher than 0.5 are considered to have roughly the same fold. *C*, magnified view of the N-terminal region of PTAR1 and Ecm9. Both proteins share a unique N-terminal extension comprising an α-helix followed by a three-stranded β-sheet. *D*, phylogenetic tree of the protein prenyltransferase α subunit family. Protein sequences from human (*H. sapiens*), mouse (*M. musculus*), zebrafish (*D. rerio*), fruit fly (*D. melanogaster*), nematode (*C. elegans*), budding yeast (*S. cerevisiae*), fission yeast (*S. pombe*), cellular slime mold (*D. discoideum*), Arabidopsis (*A. thaliana*), liverwort (*M. polymorpha*), Tetrahymena (*T. thermophila*), and sulfothermophilic red alga (*G. sulphuraria*) were analyzed using the maximum likelihood method. Predicted structures of budding yeast FNTA (Ram2), RABGGTA (Bet4), and PTAR1 homologs from Tetrahymena, Arabidopsis, budding yeast, and fruit fly are displayed. The N-terminal β-sheet of PTAR1 homologs is highlighted in *yellow*. No PTAR1 homologs were found in *C. elegans* or *S. pombe*.

To explore the possibility of Cys196 geranylgeranylation, we first searched for yeast homologs of PTAR1 using BLAST but failed to find any proteins with significant sequence homology to human PTAR1. Considering the possibility that structurally similar proteins may exist despite low sequence conservation, we performed a DALI search against the yeast AlphaFold-predicted structure library (35, 36), using the previously determined crystal structure of human PTAR1 as a query (6). This analysis identified three proteins—Ecm9, Bet4, and Ram2—with significant structural homology to human PTAR1 (Figure 2*A*). Among these, Bet4 and Ram2 are known as the α subunits of yeast GGT2 and FT/GGT1, respectively. The top-scoring protein, Ecm9, is an uncharacterized protein originally identified in a genetic screen for mutants with high sensitivity to a cell wall synthesis inhibitor (37).

Despite the low sequence conservation between human PTAR1 and Ecm9 (Fig. S1), structural alignment revealed a high degree of three-dimensional similarity, with a root mean square deviation (RMSD) of 3.94 Å (Fig. 2*B*). The template modeling (TM)-score for PTAR1 and Ecm9 was 0.73, indicating that they share roughly the same topological fold. Notably, a unique N-terminal β-sheet structure of PTAR1, which is involved in Ykt6 recognition (6), is also present in Ecm9 (Fig. 2*C*). These structural similarities suggest that Ecm9 may function as the budding yeast counterpart of human PTAR1.

Following the identification of Ecm9, we performed extensive searches for PTAR1 homologs across diverse eukaryotic species using BLAST and AlphaFold structure prediction. These analyses revealed that PTAR1 homologs are broadly conserved among eukaryotes (Fig. 2*D*). Phylogenetic analysis indicated that the prenyltransferase α subunit family proteins are classified into three clades: FNTA (FT/GGT1α), RABGGTA (GGT2α), and PTAR1 (GGT3α). Ecm9 was placed within the PTAR1 clade (Fig. 2*D*). The presence of PTAR1 homologs in fungi, plants, and protists suggested that this clade emerged early in eukaryotic evolution. Interestingly, AlphaFold-predicted structures revealed that all PTAR1 homologs possess a characteristic three-stranded β-sheet at the N-terminus, which is absent in FNTA and RABGGTA homologs. This structural conservation suggests that PTAR1 homologs share a common role in Ykt6 recognition.

### Ecm9 forms a heterodimeric complex with Bet2

**Figure 3:**
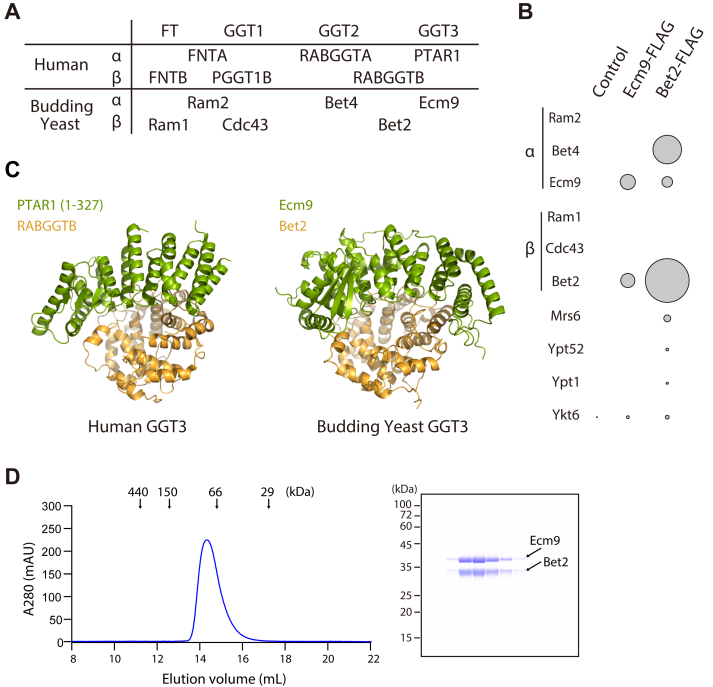
**Ecm9 and Bet2 form a heterodimeric complex.***A*, overview of protein prenyltransferases and their subunits in humans and budding yeast. FT and GGT1 share the same α subunit, whereas GGT2 and GGT3 share the same β subunit. *B*, bubble plot showing interactors of Bet2 and Ecm9. Anti-FLAG immunoprecipitates were purified from control yeast cells or cells expressing Bet2-FLAG or Ecm9-FLAG and analyzed by LC-MS/MS. Prenyltransferase subunits and associated proteins are represented as circles. The circle size is proportional to the intensity of detected proteins. *C*, structural comparison of the crystal structure of human GGT3 (PDB: 6J6X) and the AlphaFold-predicted structure of yeast GGT3. *D*, gel filtration chromatogram of recombinant yeast GGT3. Ecm9 and Bet2 were co-expressed in ExpiSf insect cells and purified using a Superdex 200 gel filtration column. The *right* panel shows SDS-PAGE analysis of the purified GGT3 complex visualized by Coomassie staining.

As shown in Figure 3*A*, four distinct prenyltransferases are formed in humans through combinations of three α subunits (FNTA, RABGGTA, PTAR1) and three β subunits (FNTB, PGGT1B, RABGGTB). In humans, FT and GGT1 share the same α subunit, while GGT2 and GGT3 share the same β subunit. If this combination pattern is conserved in yeast, Ecm9 is predicted to form a complex with Bet2, the β subunit of yeast GGT2. To test this, we inserted a FLAG tag at the C-terminus of the *ECM9* and *BET2* genes in yeast. Using an anti-FLAG antibody, we immunopurified Ecm9 and Bet2, followed by quantitative analysis of associated proteins using data-independent acquisition (DIA) mass spectrometry. As shown in Figure 3*B*, Bet2 (GGT2β) was abundantly detected in the Ecm9-FLAG immunoprecipitates, whereas Ram1 (FTβ) and Cdc43 (GGT1β) were not detected. Conversely, Ecm9 was detected in the Bet2-FLAG samples, which also contained Bet4 (GGT2α), Mrs6 (the yeast REP homolog), and the yeast Rab proteins Ypt51 and Ypt1. Ykt6 was detected in both samples above background levels. These results clearly indicate that Ecm9 specifically binds to Bet2, forming a complex distinct from GGT2. Interestingly, AlphaFold predicted the heterodimeric structure of Ecm9 and Bet2 with high confidence (Figs. 3*C*, S2). In contrast, no reliable interaction was predicted between Ecm9 and either Ram1 (FTβ) or Cdc43 (GGT1β), supporting the specificity of Ecm9 for Bet2.

To experimentally confirm the formation of the Ecm9–Bet2 heterodimer, the two proteins were co-expressed in ExpiSf insect cells and purified using column chromatography. As shown in Figure 3*D*, Ecm9 and Bet2 co-eluted at approximately 80 kDa on a Superdex 200 gel filtration column, consistent with the formation of a 1:1 stoichiometric complex. These results confirm the formation of the Ecm9–Bet2 complex in budding yeast, which is structurally analogous to the human PTAR1–RABGGTB complex.

### Yeast GGT3 transfers geranylgeranyl to mono-farnesylated Ykt6

**Figure 4:**
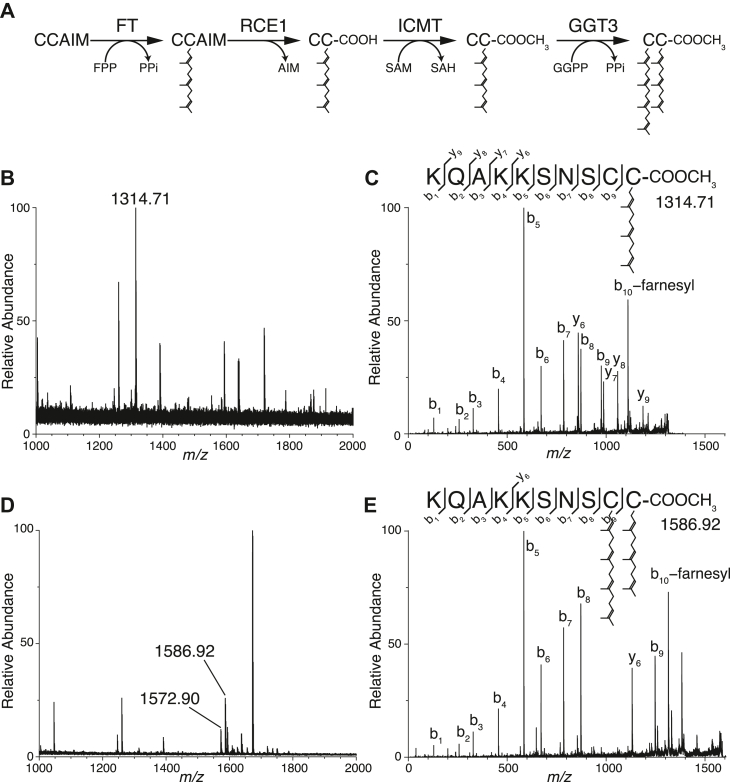
**Yeast GGT3 Geranylgeranylates Ykt6.***A*, schematic model of Ykt6 C-terminal processing in mammals. Mammalian Ykt6 undergoes a series of post-translational modifications, including farnesylation by FT, cleavage by RCE1, methylation by ICMT, and geranylgeranylation by GGT3. FPP, farnesyl pyrophosphate; PPi, pyrophosphate; SAM, S-adenosylmethionine; SAH, S-adenosylhomocysteine; GGPP, geranylgeranyl pyrophosphate. *B*, MS spectrum of a chymotryptic digest of recombinant yeast Ykt6 treated with FT, RCE1, and ICMT. Recombinant yeast Ykt6 was first farnesylated by FT, followed by C-terminal cleavage and methylation. The processed protein was digested with chymotrypsin and analyzed using MALDI-TOF mass spectrometry. The ion peak at m/z 1314.71 corresponds to the Cys197-farnesylated C-terminal peptide. *C*, MS/MS spectrum of the Cys197-farnesylated peptide. The precursor ion (m/z 1314.71) was subjected to collision-induced dissociation (CID) fragmentation and analyzed by MALDI-TOF/TOF mass spectrometry. *D*, MS spectrum of a chymotryptic digest of recombinant yeast Ykt6 treated with FT, RCE1, ICMT, and yeast GGT3. Cys197-farnesylated Ykt6 was incubated with yeast GGT3 and GGPP, then digested with chymotrypsin and analyzed by MALDI-TOF mass spectrometry. The ion peak at m/z 1586.92 represents the doubly prenylated C-terminal peptide, while the peak at m/z 1572.90 corresponds to the doubly prenylated but unmethylated C-terminal peptide. *E*, MS/MS spectrum of the doubly prenylated peptide showing geranylgeranylation at Cys196 and farnesylation at Cys197. The precursor ion (m/z 1586.92) was subjected to CID fragmentation and analyzed by MALDI-TOF/TOF mass spectrometry.

Figure 4*A* presents a schematic model of the double prenylation process of human Ykt6. First, Ykt6 undergoes farnesylation, followed by cleavage of the AIM tripeptide and subsequent C-terminal methylation. The resulting mono-farnesylated, C-terminally processed form of Ykt6 serves as an efficient substrate for human GGT3. To test whether yeast GGT3 can transfer a geranylgeranyl group to mono-farnesylated Ykt6, we performed *in vitro* prenylation reactions using recombinant proteins and analyzed the modified peptide products by MALDI-TOF/TOF mass spectrometry. Figure 4*B* shows the mass spectrum of chymotrypsin-digested peptides derived from recombinant yeast Ykt6 treated with human FT, RCE1, and ICMT. An ion peak with an m/z value of 1314.71 was detected, which closely matched the theoretical mass of the farnesylated, C-terminally processed peptide (1314.73 Da). MS/MS analysis of this precursor ion confirmed farnesylation at the C-terminal cysteine, Cys197 (Fig. 4*C*). Figure 4*D* shows the mass spectrum of Ykt6 peptides following further incubation with yeast GGT3. An ion peak with a mass shift of 272 Da relative to the farnesylated peptide was detected (m/z 1586.92), consistent with the addition of a geranylgeranyl group. MS/MS analysis of this peak confirmed the covalent attachment of a geranylgeranyl group to Cys196 (Fig. 4*E*). Additionally, we detected an ion peak corresponding to a doubly prenylated peptide with an unmethylated C-terminus (m/z 1572.90; Figs. 4*D*, S3), likely resulting from incomplete methylation by ICMT. Collectively, these results demonstrate that yeast GGT3 can transfer a geranylgeranyl group to Cys197-farnesylated Ykt6, irrespective of its C-terminal methylation, to produce doubly prenylated Ykt6.

### Ykt6 is doubly prenylated in budding yeast

**Figure 5:**
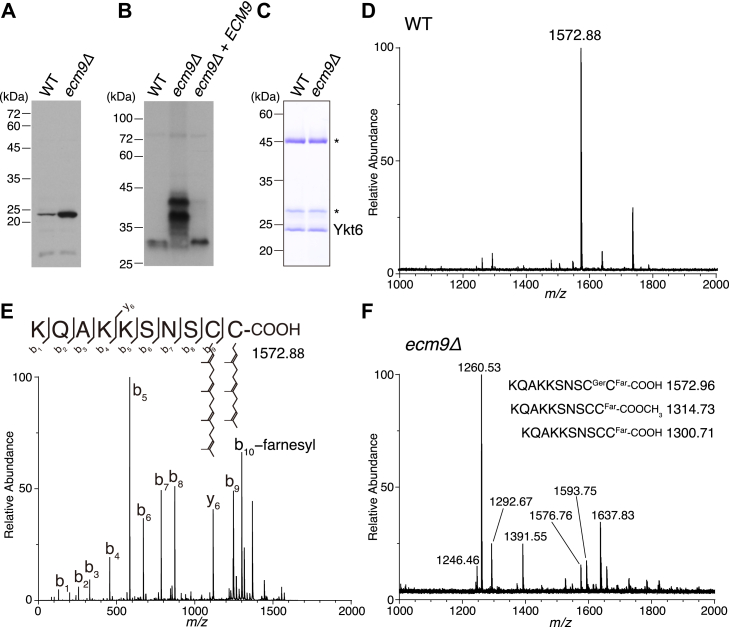
**Endogenous Ykt6 is doubly prenylated in budding yeast.***A*, immunoblot analysis of Ykt6. Cell lysates from WT and *ecm9Δ* cells (10 μg protein each) were subjected to SDS-PAGE and analyzed by immunoblotting with an anti-Ykt6 polyclonal antibody. *B*, immunoblot analysis of Ykt6 separated by DOC-PAGE. Cell lysates from WT, *ecm9Δ*, and rescued *ecm9Δ* cells were subjected to DOC-PAGE to separate prenylated forms of Ykt6, followed by immunoblotting with the anti-Ykt6 polyclonal antibody. *C*, SDS-PAGE analysis of immunopurified endogenous Ykt6. Endogenous Ykt6 was purified from the cytosol of yeast cells through ammonium sulfate precipitation, gel filtration, and immunoaffinity chromatography using an anti-Ykt6 monoclonal antibody. The purified proteins were analyzed by SDS-PAGE and visualized with Coomassie staining. Asterisks indicate the heavy and light chains of the anti-Ykt6 monoclonal antibody. *D*, MS spectrum of a chymotrypsin digest of the endogenous Ykt6 purified from WT yeast cells. Purified Ykt6 was digested with chymotrypsin, and the resulting peptides were analyzed by MALDI-TOF mass spectrometry. The ion peak at m/z 1572.88 corresponds to the C-terminal peptide of endogenous Ykt6. *E*, MS/MS spectrum of the C-terminal peptide of endogenous Ykt6 from WT yeast cells showing geranylgeranylation at Cys196 and farnesylation at Cys197. The precursor ion (m/z 1572.88) was subjected to CID fragmentation and analyzed by MALDI-TOF/TOF mass spectrometry. *F*, MS spectrum of a chymotrypsin digest of the endogenous Ykt6 purified from *ecm9Δ* yeast cells. The ion peak at theoretical m/z 1572.96, corresponding to the C-terminal peptide of doubly prenylated Ykt6, was not detected. The ion peaks at theoretical m/z 1314.73 and 1300.71, corresponding to the C-terminal peptide of mono-farnesylated Ykt6 with or without methylation, respectively, were also absent.

Next, we investigated the prenylation state of endogenous Ykt6 in budding yeast. Immunoblot analysis using a rabbit polyclonal antibody revealed that Ykt6 was overexpressed in *ecm9Δ* cells compared to wild-type (WT) cells (Fig. 5*A*). To evaluate the degree of Ykt6 prenylation, cell lysates from WT and *ecm9Δ* cells were analyzed using sodium deoxycholate-polyacrylamide gel electrophoresis (DOC-PAGE), a technique that separates prenylated proteins based on their lipid modification states (Fig. 5*B*) (6). In WT cells, Ykt6 appeared as a single band, indicating a uniform prenylation state. In contrast, Ykt6 from *ecm9Δ* cells displayed multiple bands with slower mobility, suggesting several modification states distinct from that in WT cells. When *ECM9* expression was restored, the band pattern returned to that observed in WT cells.

To confirm that budding yeast Ykt6 is doubly prenylated, we generated a rat monoclonal antibody that can efficiently immunoprecipitate budding yeast Ykt6. Using this antibody, endogenous Ykt6 was immunopurified from the cytosol of WT and *ecm9Δ* cells (Fig. 5*C*). The purified Ykt6 proteins were digested with chymotrypsin and analyzed by MALDI-TOF/TOF mass spectrometry. As shown in Figure 5*D*, an ion peak corresponding to a doubly prenylated C-terminal Ykt6 peptide was detected in the sample purified from WT cells. MS/MS spectrum of this peak unambiguously identified geranylgeranylation at Cys196 and farnesylation at Cys197 (Fig. 5*E*), whereas C-terminal methylation was not observed. Notably, this ion peak was absent in the sample purified from *ecm9Δ* cells (Fig. 5*F*). Ion peaks corresponding to other forms of C-terminal Ykt6 peptide, including mono-farnesylated ones, were also undetectable, presumably due to the low recovery of these peptides compared to doubly prenylated peptides. These results demonstrate that in budding yeast, the C-terminus of Ykt6 is constitutively modified with two prenyl groups—farnesyl and geranylgeranyl—and that Ecm9 is essential for this modification.

### Ecm9 is required for cell wall integrity

**Figure 6:**
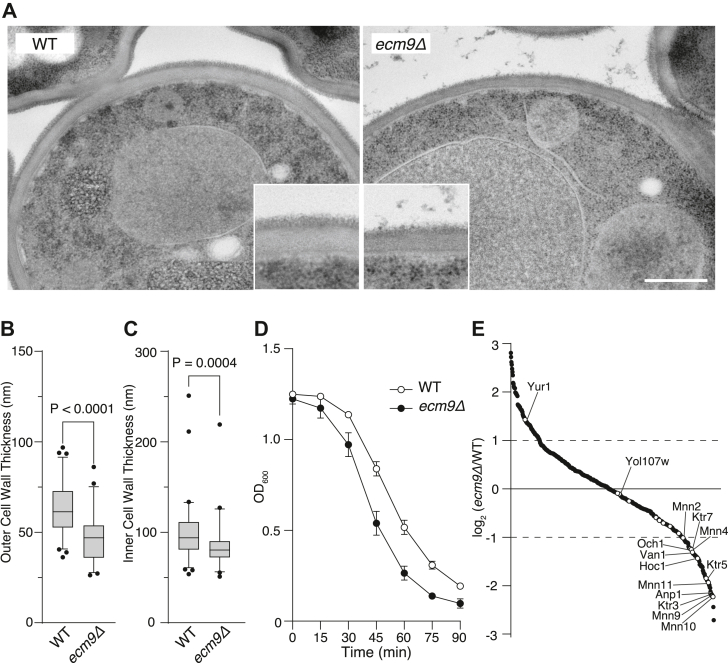
**Ecm9 is required for cell wall integrity.***A,* electron micrographs of WT and *ecm9Δ* cells. Yeast cells were grown to log phase in YPD medium and processed for electron microscopy (EM) using the rapid freezing and freeze-substitution fixation method. Insets show the cell wall at higher magnification. Scale bar, 500 nm. *B* and *C*, thickness measurements of the outer (*B*) and inner (*C*) cell wall in WT and *ecm9Δ* cells. Cell wall thickness was measured from EM images. For the outer cell wall, 69 WT and 48 *ecm9Δ* cells were analyzed. For the inner cell wall, 67 WT and 45 *ecm9Δ* cells were analyzed. Data are presented as median values with interquartile ranges. Statistical analysis was performed using the Kolmogorov–Smirnov test. *D*, zymolyase sensitivity assay. WT and *ecm9Δ* cells were grown to log phase in YPD medium and treated with 100 μg/ml zymolyase in H_2_O. Optical density at 600 nm (OD_600_) was measured at the indicated time points. Data are presented as the mean ± SEM of three independent experiments. *E*, quantitative proteomic analysis of the Golgi apparatus. The Golgi apparatus was immunopurified from WT and *ecm9Δ* cells expressing Yol107w-3×EGFP using an anti-GFP nanobody. Golgi proteins were extracted with detergent, digested, and analyzed by LC-MS/MS. A total of 590 proteins were ranked and plotted based on intensity ratios between WT and *ecm9Δ* cells. Golgi glycosyltransferases and Yol107w are indicated by *white* circles.

The *ecm9* mutant exhibits hypersensitivity to inhibitors of cell wall synthesis (37), suggesting that the mutant has an abnormality in cell wall structure. The cell wall of budding yeast consists of a bilayer structure: the outer layer is primarily composed of highly mannosylated glycoproteins known as mannoproteins, while the inner layer is composed of polysaccharides such as β-glucans and chitins (38). Electron microscopy revealed that the cell wall in *ecm9Δ* cells was significantly thinner (Fig 6, *A–C*), particularly in the outer mannoprotein layer. To evaluate cell wall integrity, we performed a zymolyase digestion assay. Under hypoosmotic conditions, yeast cells with digested cell walls rupture, resulting in a decrease in optical density. This assay showed that *ecm9Δ* cells were more susceptible to zymolyase digestion (Fig. 6*D*). These results indicate that *ecm9Δ* cells have defects in cell wall integrity, with notable structural abnormalities, particularly in the outer layer.

We next analyzed the protein composition of the Golgi apparatus using organelle immunopurification with the Golgi marker protein Yol107w, as mannoproteins undergo extensive glycosylation within the Golgi apparatus. Quantitative proteomic analysis of the purified Golgi samples revealed a significant decrease in Golgi glycosylation enzymes, including the mannosyltransferases Mnn9 and Anp1, in *ecm9Δ* cells (Fig. 6*E*). These results indicate that Ecm9 is necessary for the proper localization of Golgi-resident enzymes.

### Ecm9 is essential for membrane localization of Ykt6 and efficient autophagy

**Figure 7:**
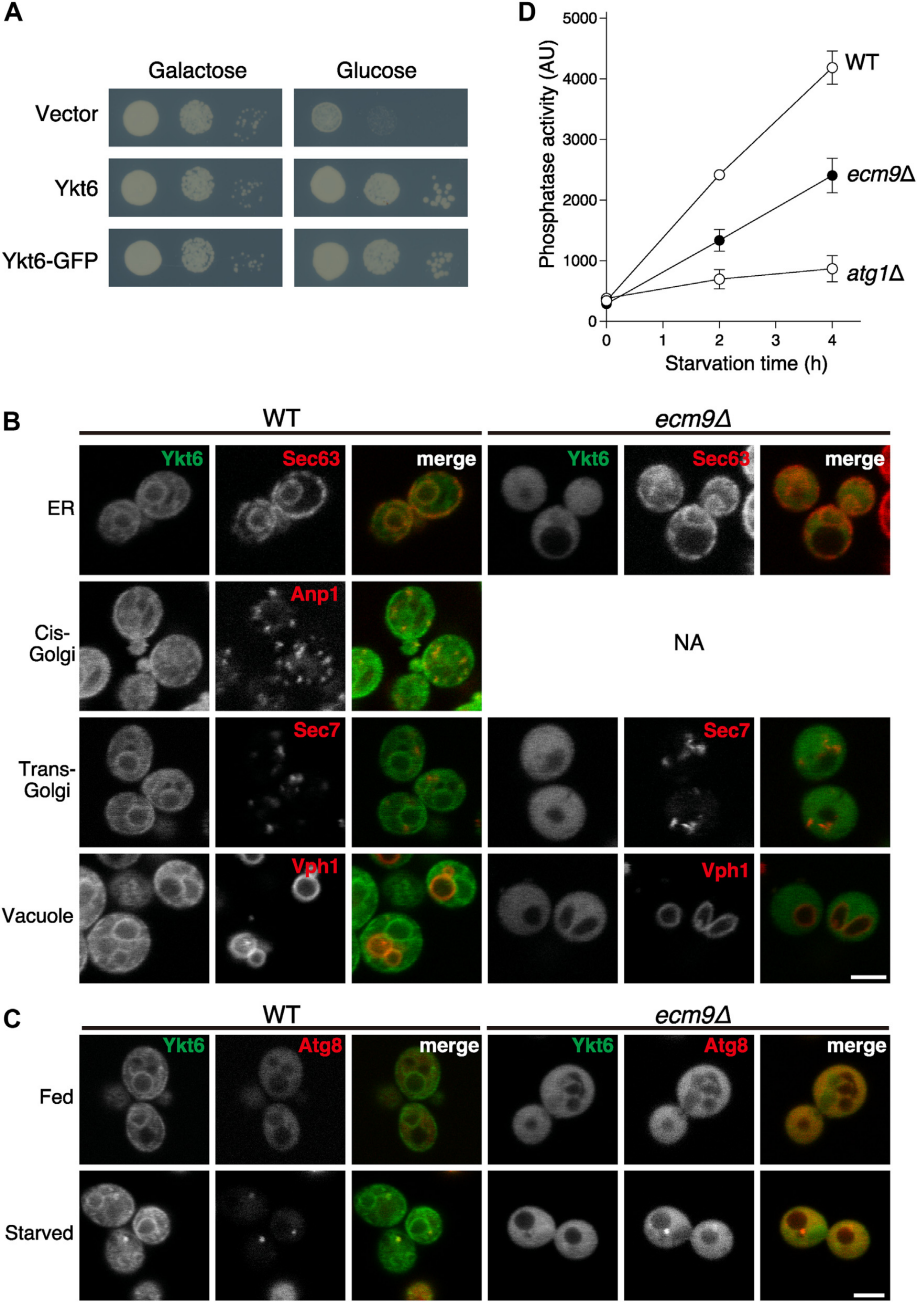
**Double prenylation of yeast Ykt6 is necessary for efficient autophagy.***A*, Ykt6-GFP suppresses lethality caused by depletion of endogenous Ykt6. A yeast strain with Ykt6 expression under the control of the *GAL1* promoter was transformed with an empty vector, a Ykt6 expression vector, or a Ykt6-GFP expression vector. Serial dilutions of the cultures were spotted onto glucose or galactose agar plates, and yeast growth was evaluated. *B*, subcellular localization of Ykt6-GFP. WT and *ecm9Δ* cells expressing Ykt6-GFP along with Sec63, Anp1, Sec7, or Vph1 tagged with 3×mCherry were grown in YPD medium and observed using confocal microscopy. In WT cells, Ykt6-GFP colocalized with Sec63 and partially with Anp1 and Vph1. In *ecm9Δ* cells, Ykt6-GFP was uniformly dispersed, and no membrane-like structures were observed. No *ecm9Δ* cells expressing Anp1-3×mCherry were obtained, possibly due to a growth defect. Scale bar, 3 μm. NA, not available. *C*, localization of Ykt6-GFP to autophagosomes under starvation conditions. The colocalization of Ykt6-GFP with mCherry-Atg8 was analyzed in cells grown in YPD medium (fed) or nitrogen-starved for 1 h (starved). In WT cells, Ykt6-GFP colocalized with mCherry-Atg8 puncta under starvation conditions, but no colocalization was observed in *ecm9Δ* cells. Scale bar, 3 μm. *D*, Pho8Δ60 assay. WT, *ecm9Δ*, and *atg1Δ* cells expressing Pho8Δ60 were nitrogen-starved for the indicated times. The cells were lysed, and autophagy-dependent activation of Pho8Δ60 was analyzed by measuring alkaline phosphatase activity. Data are presented as the mean ± SEM of three independent experiments.

To analyze the localization of Ykt6 in WT and *ecm9Δ* cells, we used GFP-tagged Ykt6. Since the N-terminus of Ykt6 directly interacts with PTAR1 in humans (6) and AlphaFold predicts that yeast Ykt6 binds Ecm9 in the same configuration as in humans (Fig. S4), an N-terminal tag may interfere with its recognition by GGT3 in yeast. To circumvent this, we employed a version of Ykt6 in which GFP was inserted into the first loop of the N-terminal longin domain (referred to as Ykt6-GFP) (39). Ykt6-GFP is functional, as its expression rescues the growth defect caused by the depletion of endogenous Ykt6 (Fig 7*A*). Figure 7*B* shows the localization patterns of Ykt6-GFP and various organelle marker proteins tagged with 3×mCherry. There were no significant differences in the localization of these markers between WT and *ecm9Δ* cells. Consistent with previous studies (29, 39), Ykt6-GFP in WT cells was primarily localized to the cytosol and ER membranes, with minor localization to vacuolar and cis-Golgi membranes. In contrast, in *ecm9Δ* cells, Ykt6-GFP was diffusely distributed throughout the cytoplasm, with no detectable membrane localization.

Ykt6 is known to mediate the fusion of autophagosomes and vacuoles. To investigate the role of Ykt6 double prenylation in autophagy, we analyzed the co-localization of Ykt6-GFP and mCherry-Atg8, an autophagosome marker. In WT cells, Ykt6-GFP co-localized with mCherry-Atg8 under nitrogen starvation conditions, indicating autophagosomal localization of Ykt6 (Fig. 7*C*). In *ecm9Δ* cells, however, Ykt6-GFP remained in the cytoplasm even under starvation conditions, and did not co-localize with mCherry-Atg8.

To assess autophagy activity, we used the truncated form of Pho8 phosphatase, Pho8Δ60 (40). Under nutrient-rich conditions, Pho8Δ60 remains in the cytosol, but under starvation conditions, it translocates to the vacuole and is activated in an autophagy-dependent manner. WT, *ecm9Δ*, and *atg1Δ* cells expressing Pho8Δ60 were nitrogen-starved, and phosphatase activity was measured. Autophagy-dependent phosphatase activity in *ecm9Δ* cells was reduced to approximately 60% of that in WT cells (Fig. 7*D*). These results indicate that double prenylation is essential for the autophagosomal localization of Ykt6 and efficient autophagy.

## Discussion

In this study, we employed structural predictions to identify budding yeast Ecm9 as the structural homolog of human PTAR1. Ecm9 functions as the α subunit of yeast GGT3 and catalyzes the double prenylation of Ykt6. Phylogenetic and structural analyses indicate that PTAR1 homologs are widely conserved among eukaryotes, including fungi, plants, and protists. This conservation suggests that double prenylation is a common post-translational modification of Ykt6 across eukaryotic species. Previous studies in mammals and budding yeast proposed a mechanistic model in which Ykt6 activity is regulated by reversible palmitoylation (23, 33). However, this model was based on experiments using N-terminally tagged Ykt6, which likely detected palmitoylation as a compensatory modification. Our data, together with prior findings (6), demonstrate that endogenous Ykt6 is doubly prenylated in both mammals and budding yeast. Since protein prenylation is an irreversible process, the previous model of reversible palmitoylation as a regulatory mechanism for Ykt6 is unlikely.

Recent advances in synthetic chemistry have enabled the comprehensive identification of prenylated proteins through metabolic labeling with functionalized prenyl lipid probes (41, 42). However, direct detection of prenylated peptides remains technically challenging due to their low abundance and high hydrophobicity (43). Therefore, the analysis of natively prenylated peptides requires the purification of sufficient amounts of prenylated proteins. In this study, we immunopurified Ykt6 from yeast cells and successfully determined its C-terminal prenyl modification using MALDI-TOF/TOF mass spectrometry. Our data provide the first direct and definitive evidence for double prenylation of endogenous Ykt6. To date, Ykt6 is the only protein known to undergo double prenylation with farnesyl and geranylgeranyl groups.

Yeast growth assays revealed that the Ykt6^C196S^ mutant fails to support the growth of Ykt6-depleted yeast cells. In contrast, *ecm9Δ* cells grow normally despite the absence of geranylgeranylation at Cys196. These observations suggest that alternative lipid modifications at Cys196 may compensate for the lack of geranylgeranylation in *ecm9Δ* cells. Although we were unable to determine the exact lipid modification states of Ykt6 in *ecm9Δ* cells, DOC-PAGE analysis indicates that Ykt6 exists in multiple lipid-modified forms in these cells (Fig. 5*B*). While these compensatory modifications are insufficient to fully restore Ykt6 function, they may provide the minimal activity required for the survival of *ecm9Δ* cells. Additionally, overexpression of Ykt6 may also contribute to the viability of *ecm9Δ* cells.

Although this study reveals that PTAR1 homologs are more widely conserved than previously recognized, we could not identify homologous genes for PTAR1 in nematodes or fission yeast. However, the presence of PTAR1 homologs in plants and protists, which are thought to have diverged earlier in evolution (44), suggests a lineage-specific deletion of the PTAR1 gene in nematodes and fission yeast. The conservation of the C-terminal tandem cysteine motif in these species implies the possibility that GGT2, which shares the same catalytic β subunit with GGT3, may have adapted to fulfill the role of GGT3 in these species. Alternatively, Ykt6 might undergo distinct lipid modifications functionally equivalent to double prenylation. Direct analysis of the Ykt6 C-terminal peptide in nematodes and fission yeast will be necessary to resolve this issue.

*ECM9* was originally identified in a genetic screen for budding yeast mutants (**e**xtra**c**ellular **m**utants) that exhibit high sensitivity to the cell wall synthesis inhibitor calcofluor white (37). Electron microscopy revealed that *ecm9Δ* cells have thinner cell walls, particularly in the outer mannoprotein layer. Since mannoproteins undergo extensive mannosylation in the Golgi apparatus, these findings suggest defects in mannosyltransferase function in the Golgi of *ecm9Δ* cells. Interestingly, tagging the cis-Golgi mannosyltransferase Anp1 with 3×mCherry in *ecm9Δ* cells caused synthetic lethality, preventing the observation of Anp1 localization. A similar defect was observed with Mnn9, another cis-Golgi mannosyltransferase, suggesting a functional relationship between Ecm9 and these Golgi-localized mannosyltransferases. Since the localization of Golgi glycosyltransferases is maintained by intra-Golgi retrograde trafficking (45, 46), the loss of double prenylation of Ykt6 may lead to their mislocalization. Consistently, Golgi proteome analysis revealed a reduction in most Golgi-localized glycosyltransferases in *ecm9Δ* cells. In humans, PTAR1 knockout cells exhibit defects in LAMP1 sialylation in the Golgi (6), highlighting the critical role of Ykt6 double prenylation in maintaining Golgi glycosyltransferase functions in both yeast and humans.

Unlike other SNAREs, Ykt6 dynamically shuttles between the cytosol and organelle membranes. In the cytosol, Ykt6 adopts a closed, inactive conformation and is solubilized by sequestering its prenyl groups in a hydrophobic groove (26). Our mass spectrometry analysis revealed that Ykt6 purified from yeast cytosol is doubly prenylated, indicating that Ykt6 can adopt a closed conformation by sequestering both prenyl groups. To localize to membranes, Ykt6 must switch to an open conformation and insert its prenyl groups into the lipid bilayer. In our analysis, Ykt6-GFP was localized to the cytosol and organelle membranes in WT cells. In contrast, in *ecm9Δ* cells, Ykt6-GFP was distributed in the cytoplasm with no detectable membrane localization (Fig. 7*B*). These results indicate that modification by two prenyl groups is necessary for Ykt6 to switch between open and closed states. Although the reason why Ykt6 cannot adopt an open conformation in *ecm9Δ* cells remains unclear, structural analysis suggests that mono-farnesylated Ykt6 is restricted to a tightly closed conformation, with the farnesyl group fitting snugly into the hydrophobic groove (26, 27). The second prenylation may loosen the autoinhibitory conformation of Ykt6 to provide the structural flexibility required for its transition between open and closed states. Biophysical analyses such as single-molecule FRET and molecular dynamics simulations (47, 48) of doubly prenylated Ykt6 would help elucidate the effect of double prenylation on its conformational equilibrium. While phosphorylation has been proposed to play a role in the conformational regulation of Ykt6 (28, 29, 30, 31, 32), the molecular mechanisms underlying its transition to an open conformation and subsequent membrane association remain poorly understood. Elucidating the mechanisms that control the conformational changes of doubly prenylated Ykt6 is essential for a deeper understanding of its regulatory functions.

In summary, we have demonstrated that Ecm9 functions as the α subunit of GGT3 in budding yeast and is essential for the double prenylation of Ykt6. The loss of Ykt6 double prenylation leads to cell wall fragility and defects in autophagy. Our results indicate that double prenylation is a fundamental feature of Ykt6, enabling its dynamic localization and functional versatility in intracellular membrane trafficking.

## Experimental procedures

### Reagents

Farnesyl pyrophosphate (FPP) was purchased from Isoprenoids. Geranylgeranyl pyrophosphate (GGPP) was from Cayman Chemical. EDTA-free cOmplete protease inhibitor cocktail and sequence-grade chymotrypsin were from Roche. Dithiothreitol (DTT), sodium deoxycholate (DOC), and hygromycin B were from Fujifilm Wako Chemicals. YPD broth, yeast nitrogen base without amino acids, yeast nitrogen base without amino acids and ammonium sulfate, yeast synthetic drop-out media supplements without uracil, S-adenosylmethionine (SAM), α-cyano-4-hydroxycinnamic acid (CHCA), Angiotensin II, ACTH fragment 18 to 39, and concanavalin A were from Sigma Aldrich. Glutathione Sepharose 4B, and PreScission protease were from Cytiva. Ni-NTA agarose was from Qiagen. Zymolyase 20 T was from Nacalai Tesque. Nourseothricin was from Jena Bioscience. Dynabeads protein G magnetic beads were from Thermo Scientific. All other chemicals and general reagents were obtained from Wako or Sigma.

### Yeast strains and constructs

Yeast strains used in this study are listed in Table S1. All the strains were constructed in the BY4741 genetic background. Yeast genome modification was performed by homologous recombination, using linearized plasmids or PCR-amplified DNA fragments. The 3′ coding sequences of *SEC63*, *ANP1*, *SEC7*, and *VPH1* were amplified by PCR using BY4741 genomic DNA as a template and cloned into pBluescript CaURA3 (49) with a C-terminal 3×mCherry tag. Similarly, the 3′ coding sequence of *YOL107w* was cloned with a C-terminal 3×EGFP tag, and the 3′ coding sequences of *BET2* and *ECM9* were cloned with a C-terminal FLAG tag. These plasmids were linearized at a restriction enzyme site located within the 3′ coding regions and used for transformation. The yeast strain expressing *YKT6* under the control of the *GAL1* promoter was generated by PCR-based homologous recombination. The kanMX6-PGAL1 cassette was amplified from pFA6-kanMX6-PGAL1 (Addgene #41605) (50) using primers flanking the *YKT6* promoter and used for transformation. Yeast strains expressing *pho8Δ60* were generated as described (40). Successful transformants were selected on appropriate selection agar plates and verified by colony PCR and sequencing.

The coding sequence of *YKT6* was amplified by PCR from BY4741 genomic DNA. The cysteine-to-serine mutants (Cys196Ser, Cys197Ser, and Cys196/197Ser) were generated *via* PCR-based site-directed mutagenesis. The *YKT6-GFP* construct was created by inserting the mEGFP sequence between Gly13 and Glu14 of *YKT6*. These DNAs were cloned into a modified CEN plasmid (pRS316) containing the *YKT6* promoter region (242 bases upstream of the initiation codon) with the nourseothricin resistance cassette natNT2 (51). The mCherry-ATG8 expression vector was constructed by replacing the GFP tag and CaURA3 selection marker of CuGFP-ATG8(416) (Addgene #49423) with mCherry and the hygromycin resistance cassette hphNT1 (51), respectively. For *ECM9* rescue, the *ECM9* coding sequence was cloned under the *CYC1* promoter into pRS316 to achieve moderate expression.

### Recombinant protein expression and purification

For the purification of recombinant proteins from *E. coli*, the coding sequences of full-length Ykt6 and the longin domain of Ykt6 (residues 1–139) were amplified by PCR from BY4741 genomic DNA and subcloned into the NdeI-XhoI site of the bacterial expression vector pRSET (Invitrogen). Recombinant Ykt6 was overexpressed in BL21(DE3) cells and purified using ammonium sulfate precipitation, hydrophobic interaction chromatography, and gel filtration chromatography as previously described (6).

Recombinant yeast GGT3 was expressed in ExpiSf insect cells. The coding sequence of *ECM9* was synthesized with codon optimization (Integrated DNA Technologies) and cloned into pFastBac1 (Invitrogen). The coding sequence of *BET2* was cloned into pFastBac1 with an N-terminal GST tag. Recombinant baculoviruses were produced using the Bac-to-Bac baculovirus expression system. ExpiSf cells were co-infected with baculoviruses encoding *ECM9* and *GST-BET2* according to the manufacturer’s instructions. Seventy-two hours after infection, the cells were harvested and resuspended in 80 ml of buffer A (50 mM HEPES-KOH pH 7.4, 50 mM NaCl, 5 mM MgCl_2_, 1 mM DTT) supplemented with a protease inhibitor cocktail. The cells were disrupted by sonication, and the suspension was centrifuged at 100,000*g* for 1 h. The supernatant was incubated with glutathione Sepharose 4B beads at 4 °C for 2 h. The beads were washed with buffer A, and bound proteins were eluted with buffer A containing 10 mM glutathione. The eluate was desalted using a PD-10 column. The GST tag was cleaved by PreScission protease at 4 °C overnight, and cleaved GST was removed using a glutathione Sepharose 4B column. GGT3 was further purified using MonoQ and Superdex 200 Increase columns in buffer A. The purified protein was aliquoted and stored at −80 °C until use. Recombinant human FT and ExpiSf membrane fractions containing human RCE1 and ICMT were produced as previously described (6).

### Antibodies

The anti-Ykt6 monoclonal antibody was generated by immunizing Wistar rats with a mixture of the recombinant full-length and longin domains of Ykt6. The antibody was purified from hybridoma culture supernatants using ammonium sulfate precipitation and gel filtration chromatography. Anti-Ykt6 polyclonal antibodies were produced by immunizing rabbits with the mixture of recombinant Ykt6, followed by purification using an antigen-coupled affinity column. The anti-DYKDDDDK tag antibody (1E6) was purchased from Fujifilm Wako Chemicals. Horseradish peroxidase (HRP)-labeled anti-rabbit IgG secondary antibody was obtained from Jackson ImmunoResearch. For the production of anti-GFP and anti-mCherry nanobodies, the cDNAs encoding these nanobodies were amplified from pGEX6P1 anti-GFP nanobody (Addgene #61838) and pGEX6P1 anti-mCherry nanobody (Addgene #70696), respectively. These cDNAs were subcloned into pMES4 (Addgene #98223) with C-terminal His- and FLAG-tags. Nanobodies were purified from the periplasm of *E. coli* using Ni-NTA agarose and gel filtration as described (52).

### Homology search and phylogenetic analysis

Homology searches were performed using BLAST, DALI (36), and FoldSeek (53). For BLAST, the full-length amino acid sequence of human PTAR1 was used as the query. For DALI and FoldSeek, the crystal structure of PTAR1 from PDB: 6J6X served as the query. Structural homology was assessed using TM-align in the pairwise structural alignment tool on RCSB PDB (54). For phylogenetic analysis, 35 protein prenyltransferase α subunit sequences from various species were retrieved from UniProt. Evolutionary analyses were conducted using MEGA11 (55), with the dataset aligned using MUSCLE. The evolutionary history of the protein prenyltransferase α subunit family was inferred using the maximum likelihood method.

### Purification of binding proteins for Ecm9 and Bet2

Yeast cells expressing Bet2-FLAG and Ecm9-FLAG were grown to log phase in 200 ml of YPD. Cells were harvested by centrifugation and resuspended in 3 ml of buffer B (50 mM HEPES-KOH pH 7.4, 100 mM NaCl, 5 mM MgCl_2_, and 1 mM DTT) supplemented with a protease inhibitor cocktail. Cells were disrupted by vortexing with 6 g of zirconia beads (0.5 mm, Hira Ceramics) at 4 °C for 1 min, followed by cooling on ice for 2 min. This cycle was repeated three times. The cytosolic fraction was obtained by ultracentrifugation at 200,000*g* for 20 min. FLAG-tagged proteins were isolated using anti-FLAG antibody bound to protein G magnetic beads. The purified proteins were processed for LC-MS/MS analysis.

### Golgi proteome analysis

Yeast cells expressing Yol107w-3×EGFP were grown to log phase in 200 ml of YPD medium. The cells were treated with 0.2 g/L zymolyase in 0.2×YPD supplemented with 1 M sorbitol. Spheroplasts were collected by centrifugation, washed, and resuspended in 2 ml of buffer C (10 mM HEPES-KOH pH 7.4 and 200 mM sorbitol), and were disrupted by filtration through polycarbonate membranes with a pore size of 3.0 μm (Merck Millipore). The suspension was centrifuged twice at 500*g* for 10 min. Yol107w-3×EGFP-containing organelles were immunoprecipitated using FLAG-tagged anti-GFP nanobody bound to protein G magnetic beads *via* anti-FLAG antibody. Anti-mCherry nanobody was used as a control. The beads were washed, and the precipitated proteins were eluted with buffer C containing 1% (w/v) Triton X-100, and the samples were processed for LC-MS/MS analysis. Proteins showing more than twice the intensity of the control in WT cells were included in the analysis.

### LC-MS/MS analysis

Immunopurified samples were digested and purified using the PreOmics iST kit according to the manufacturer’s instructions. Peptides were analyzed on an Orbitrap Fusion mass spectrometer (Thermo Scientific) coupled to a nano flow high-performance liquid chromatography system (EASY-nLC 1000, Thermo Scientific) as previously described (56). A total of 500 ng of peptides was loaded onto a capillary C18 column (75 μm inner diameter × 12.5 cm, Nikkyo Technos) and separated using a 80-min gradient program with eluents A (0.1% formic acid in water) and B (0.1% formic acid in 80% acetonitrile) at a constant flow rate of 300 nl/min. The gradient program was as follows: 5% B for 1 min, 5 to 40% B over 60 min, 40 to 95% B over 2 min, and 95% B for 17 min. Eluted peptides were ionized and introduced into the mass spectrometer. Data were acquired in data-independent acquisition (DIA) mode. MS1 spectra were recorded in the Orbitrap mass analyzer at a resolution of 60,000, and peptide precursor ions were fragmented by high-energy collision-induced dissociation (HCD) to generate MS2 spectra. DIA data were processed using DIA-NN (57). All proteins detected in the DIA analysis are listed in Tables S2 and S3.

### Generation and purification of recombinant prenylated Ykt6

To generate Cys197-farnesylated, C-terminally cleaved, and methylated form of Ykt6, 10 μg of purified yeast Ykt6 was mixed with 10 μg of FT, 2 μmol of FPP, ExpiSf membranes containing recombinant RCE1 and ICMT (60 μg and 240 μg total protein, respectively), and 50 nmol of SAM in buffer A supplemented with 20 μM ZnCl_2_. The mixture was incubated at 37 °C for 30 min. Membranes were removed by ultracentrifugation at 200,000*g* for 30 min. The farnesylated Ykt6 was immunopurified using anti-Ykt6 monoclonal antibody coupled to protein G magnetic beads and processed for MALDI-TOF/TOF mass spectrometry analysis. To produce doubly prenylated Ykt6, 6 μg of yeast GGT3 and 1 μmol of GGPP were added to the membrane-free mixture and incubated for an additional 30 min at 37 °C. The doubly prenylated Ykt6 was subsequently immunopurified and subjected to MALDI analysis.

### Purification of endogenous cytosolic Ykt6

Yeast strains were cultured in 30 ml of YPD medium, and confluent precultures were diluted into 2.4 L of YPD and grown at 30 °C for 6 h. Cells were harvested, snap-frozen, and stored at −80 °C. The pellets were thawed, resuspended in 30 ml of buffer B supplemented with cOmplete protease inhibitor cocktail, and disrupted by vortexing with 45 g of zirconia beads at 4 °C for 1 min, followed by cooling on ice for 2 min. This cycle was repeated four times. The suspension was centrifuged at 100,000*g* for 1 h, and the resulting supernatant was treated with ammonium sulfate to 60% saturation and incubated on ice for 30 min with stirring. The suspension was centrifuged at 7000*g* for 20 min, and the precipitate was dissolved in 5 ml of buffer B, then applied to a HiLoad 16/600 Superdex 200 column pre-equilibrated with buffer B. Fractions containing Ykt6 were pooled and concentrated using an Amicon Ultra centrifugal filter, and Ykt6 was immunoprecipitated with anti-Ykt6 monoclonal antibody bound to protein G magnetic beads. Immunopurified Ykt6 was eluted with 100 mM glycine-HCl pH 2.0, neutralized with 3 M Tris-HCl pH 8.0, and processed for MALDI analysis.

### MALDI-TOF/TOF mass spectrometry

Purified Ykt6 samples were digested with 0.25 μg of chymotrypsin at 37 °C overnight. Digested peptides were purified using ZipTip C18 columns (Merck Millipore), and directly spotted on a MALDI plate with 5 mg/ml CHCA in 80% (v/v) acetonitrile and 0.1% (v/v) trifluoroacetic acid. MALDI-TOF/TOF analysis was performed using a TOF/TOF 5800 mass spectrometer (AB Sciex). Angiotensin II and ACTH fragment 18 to 39 were used as calibration standards.

### Immunoblot analysis of endogenous Ykt6

Yeast cells in log phase were collected by centrifugation, washed, and resuspended with 500 μl of buffer B supplemented with a protease inhibitor cocktail. Cells were disrupted by vortexing with 2 g of zirconia beads as described above. The cytosolic fraction was obtained by ultracentrifugation at 200,000*g* for 20 min. Protein samples (10 μg total) were analyzed by SDS-PAGE and DOC-PAGE followed by immunoblotting as previously described (6).

### Electron microscopy analysis

WT and *ecm9Δ* cells were grown to log phase in YPD medium. Electron microscopy was performed by Tokai Electron Microscopy using the rapid freezing and freeze-fixation method.

### Zymolyase sensitivity assay

Yeast cells were grown to log phase in 25 ml of YPD medium. Cells were harvested, washed with H_2_O, and resuspended in 25 ml of H_2_O containing 2.5 mg of zymolyase 20T. The cells were incubated at 30 °C with shaking at 225 rpm. Optical density at 600 nm (OD_600_) was measured every 15 min using a NanoPhotometer NP80 (Implen).

### Live cell imaging

For imaging of yeast cells expressing Ykt6-GFP and organelle marker proteins, yeast strains were grown to log phase in YPD medium containing 100 μg/ml nourseothricin. For imaging of yeast cells expressing mCherry-Atg8 and Ykt6-GFP, yeast strains were grown to log phase in YPD medium containing 200 μg/ml hygromycin, 100 μg/ml nourseothricin, and 0.25 mM CuSO_4_. To induce autophagy, the cells were transferred to nitrogen-deprived synthetic minimal medium (SD−N, 0.17% yeast nitrogen base without amino acids or ammonium sulfate, supplemented with 2% glucose) containing 0.25 mM CuSO_4_ and cultured for 1 h. Yeast cells were mounted on a glass-bottom chamber (Matsunami Glass) coated with concanavalin A and imaged using a Leica TCS SP8 confocal microscope.

### Pho8Δ60 assay

The Pho8Δ60 assay was performed as previously described (40). Yeast strains were grown to log phase in YPD medium, then washed and resuspended in SD−N. After 2 h or 4 h of incubation at 30 °C to induce autophagy, the cells were harvested, snap-frozen, and stored at −80 °C. The cell pellets were thawed, resuspended in buffer D (100 mM Tris-HCl pH 9.0, 10 mM MgSO_4_, and 10 μM ZnSO_4_), and disrupted by vortexing with zirconia beads at 4 °C. The suspension was centrifuged at 20,000*g* for 3 min, and the supernatant was collected. Protein concentration in the supernatant was measured, and 20 μg of protein from each sample was diluted to 0.36 ml with buffer D. The assay was initiated by adding 0.04 ml of 55 mM α-naphthyl phosphate disodium, and the mixture was incubated at 30 °C for 20 min. The reaction was stopped by adding 0.4 ml of 2 M glycine-NaOH pH 11.0. Fluorescence was measured at 345 nm for excitation and 472 nm for emission using a SpectraMax M2e fluorescent plate reader (Molecular Devices).

## Data availability

All data are in the manuscript.

## Supporting information

This article contains supporting information.

## Conflict of interests

The authors declare that they have no conflicts of interest with the contents of this article.
