# Supplementary material for: Double prenylation of budding yeast Ykt6 regulates cell wall integrity and autophagy
Source: J Biol Chem. 2025 Mar 4;301(4):108384. doi: 10.1016/j.jbc.2025.108384 (PMC12001115; doi:10.1016/j.jbc.2025.108384)
Supplement: Table S1 [file mmc1.docx]

**Table S1. Yeast strains used in this study**

| **Strain** | **Genotype** | **Reference** |
| --- | --- | --- |
| BY4741 | *MATa; his3Δ1 leu2Δ0 met15Δ0 ura3Δ0* | Euroscarf |
| Y07565 | *MATa: his3Δ1 leu2Δ0 met15Δ0 ura3Δ0 ecm9Δ::kanMX4* | Euroscarf |
| Y04547 | *MATa; his3Δ1 leu2Δ0 met15Δ0 ura3Δ0 atg1Δ::kanMX4* | Euroscarf |
| MTY27 | *kanMX6::P_GAL1_-YKT6* | This study |
| MTY28 | MTY27; *pRS316* | This study |
| MTY29 | MTY27; *pRS316-YKT6* | This study |
| MTY33 | MTY27; *pRS316- ykt6^C196S^* | This study |
| MTY34 | MTY27; *pRS316- ykt6^C197S^* | This study |
| MTY35 | MTY27; *pRS316- ykt6^C196S, C197S^* | This study |
| MTY89 | *ECM9::ECM9-FlAG* | This study |
| MTY90 | *BET2::BET2-FLAG* | This study |
| MTY68 | Y07565; *pRS316-CYC1pr-ECM9* | This study |
| MTY85 | *YOL107w:: YOL107w-3×EGFP* | This study |
| MTY86 | Y07565 *YOL107w:: YOL107w-3×EGFP* | This study |
| MTY65 | MTY27; *pRS316-YKT6-mEGFP* | This study |
| MTY47 | *ANP1::ANP1-3×mCherry pRS316 -YKT6-mEGFP* | This study |
| MTY48 | *SEC7::SEC7-3×mCherry pRS316-YKT6-mEGFP* | This study |
| MTY49 | *SEC63::SEC63-3×mCherry pRS316-YKT6-mEGFP* | This study |
| MTY50 | *VPH1:: VPH1-3×mCherry pRS316-YKT6-mEGFP* | This study |
| MTY94 | Y07565; *SEC7:: SEC7-3×mCherry pRS316-YKT6-mEGFP* | This study |
| MTY95 | Y07565; *SEC63::SEC63-3×mCherry pRS316-YKT6-mEGFP* | This study |
| MTY96 | Y07565; *VPH1:: VPH1-3×mCherry pRS316-YKT6-mEGFP* | This study |
| MTY45 | *pRS416-CUP1pr-mCherry-ATG8 pRS316-YKT6-mEGFP* | This study |
| MTY46 | Y07565; *pRS416-CUP1pr-mCherry-ATG8 pRS316-YKT6-mEGFP* | This study |
| MTY22 | *PHO8::pho8Δ60* | This study |
| MTY23 | Y07565; *PHO8::pho8Δ60* | This study |
| MTY24 | Y04547; *PHO8::pho8Δ60* | This study |
