# Supplementary material for: Double prenylation of budding yeast Ykt6 regulates cell wall integrity and autophagy
Source: J Biol Chem. 2025 Mar 4;301(4):108384. doi: 10.1016/j.jbc.2025.108384 (PMC12001115; doi:10.1016/j.jbc.2025.108384)
Supplement: Figure S1 [file mmc4.pdf]

```
PTAR1     1 MAETSEEVAVLVQRVVKDITNAFRRNPHIDEIGLIPCPEARYNRSPIVLVENKLGVESWC
Ecm9      1 MQSSLPLCREFFEKITAYLDYHDFRLTITANQPSITLPYYVDEK----AHSIELIIFKTT

PTAR1    61 VKFLLPYVHNKLLLYRTRKQWLNRDELIDVTCTLLLLNPDFTTAWNVRKELILSGTLNPI
Ecm9     57 FLSLFQEAHTYFNKTFSDQSGISNENIYYMTVGFLLTTPENKTVYNVHEDLLKR------

PTAR1   121 KDLHLGKLALTKFPKSPETWIHRRWVLQQLIQETSLPSFVTKGNLGTIPTERAQRLIQEE
Ecm9    111 -YFQDNSVLVIPDLLVKEVRLIQRLLCSSNNRINKSSSLWILYRKLFVLSLDANTLVLPD

PTAR1   181 MEVCGEAAG-RYPSNYNAWSHRIWVLQHLAKLDVKILLDELSSTKHWASMHVSDHSGFHY
Ecm9    170 ILFVFHSSGSQHFSNYYCWNTARWFYDNLP---YNKRIELFNLTKRFCFQNVKDCS----

PTAR1   240 RQFLLKSLISQTVIDSSVMEQNPLRSEPALVPPKDEEAAVSTEEPRINLPHLLEEEVEFS
Ecm9    223 SWSALAYMVCQQEEKKTDNIRDFQRLTSSFNVPFKINKVDLNFQVQPADAFTQELVKWID

PTAR1   300 TDLIDSYPGHETLWCHRRHIFYLQHHLNAGSQLSQAMEVDGLNDSSKQGYSQETKRLKRT
Ecm9    283 RTYAADWP---PYLCLLQITKFNITLRIEMDSVLLTWRNEILNFEENSGHIKMINNTPIV

PTAR1   360 PVPDSLGLEMEHRFIDQVLSTCRNVEQARFASAYRKWLVTLSQ
Ecm9    340 PEKFSNDLLTSVNFAHFGYKKLFLNKFLDKNKKEQSDS-----
```

Figure S1. Sequence alignment of human PTAR1 and Ecm9.

The amino acid sequences of human PTAR1 (UniProt: Q7Z6K3) and budding yeast Ecm9 (UniProt: Q02202) were aligned using ClustalW. Identical amino acids are shown in black, while similar amino acids are shown in gray.
