# Supplementary material for: Double prenylation of budding yeast Ykt6 regulates cell wall integrity and autophagy
Source: J Biol Chem. 2025 Mar 4;301(4):108384. doi: 10.1016/j.jbc.2025.108384 (PMC12001115; doi:10.1016/j.jbc.2025.108384)
Supplement: Figure S2 [file mmc5.pdf]

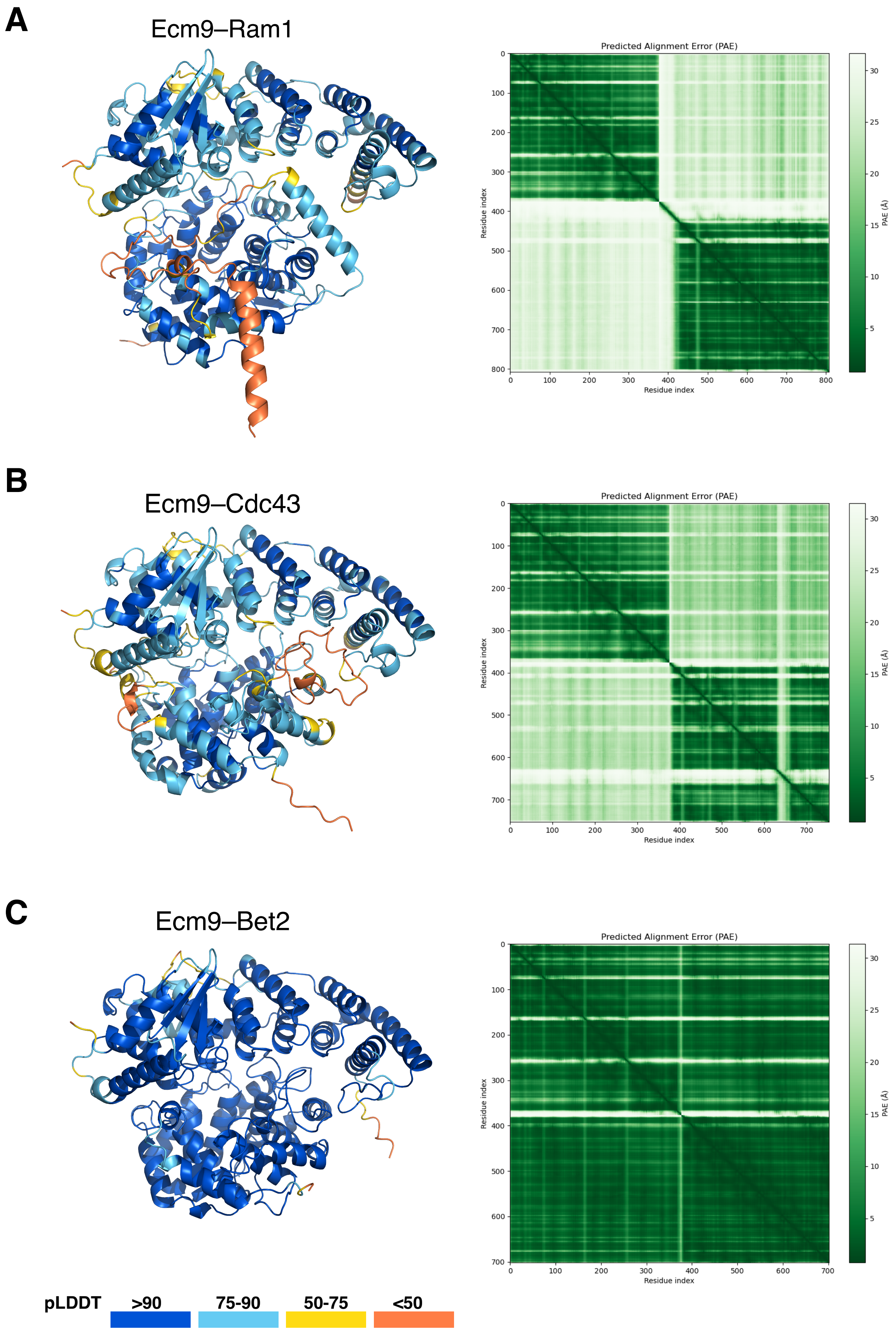

Figure S2. Predicted structures of prenyltransferase β subunits complexed with Ecm9.

Structures of Ram1, Cdc43, and Bet2 complexed with Ecm9 were predicted using AlphaFold 3 (A–C). The left panels show the predicted structures colored by the predicted local distance difference test (pLDDT) scores, and the right panels display the predicted alignment error (PAE). The predicted structures of Ram1 and Cdc43 complexed with Ecm9 exhibit larger PAEs compared to the structure of Bet2 complexed with Ecm9.
