# Supplementary material for: Double prenylation of budding yeast Ykt6 regulates cell wall integrity and autophagy
Source: J Biol Chem. 2025 Mar 4;301(4):108384. doi: 10.1016/j.jbc.2025.108384 (PMC12001115; doi:10.1016/j.jbc.2025.108384)
Supplement: Figure S3 [file mmc6.pdf]

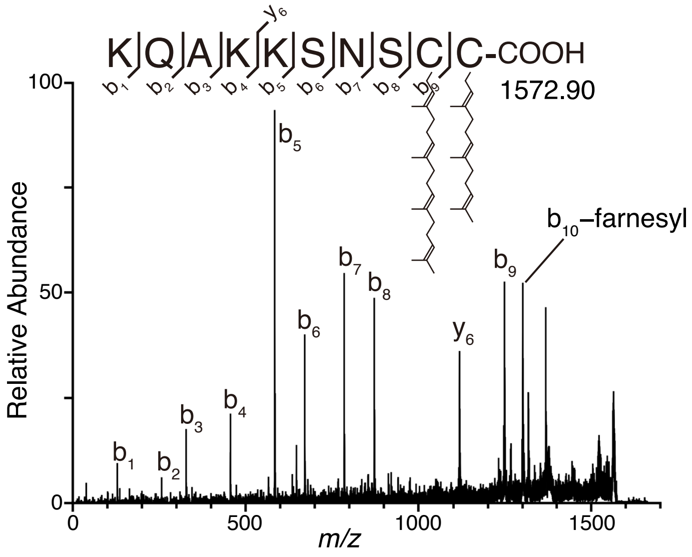

Figure S3. MS/MS analysis of doubly prenylated recombinant Ykt6 without C-terminal methylation. MS/MS spectrum of the C-terminal peptide of doubly prenylated, but not methylated, recombinant Ykt6. The precursor ion peak at m/z 1572.90 (Fig. 4D) was fragmented by CID and analyzed by MALDI-TOF/TOF mass spectrometry.
