# Supplementary material for: Double prenylation of budding yeast Ykt6 regulates cell wall integrity and autophagy
Source: J Biol Chem. 2025 Mar 4;301(4):108384. doi: 10.1016/j.jbc.2025.108384 (PMC12001115; doi:10.1016/j.jbc.2025.108384)
Supplement: Figure S4 [file mmc7.pdf]

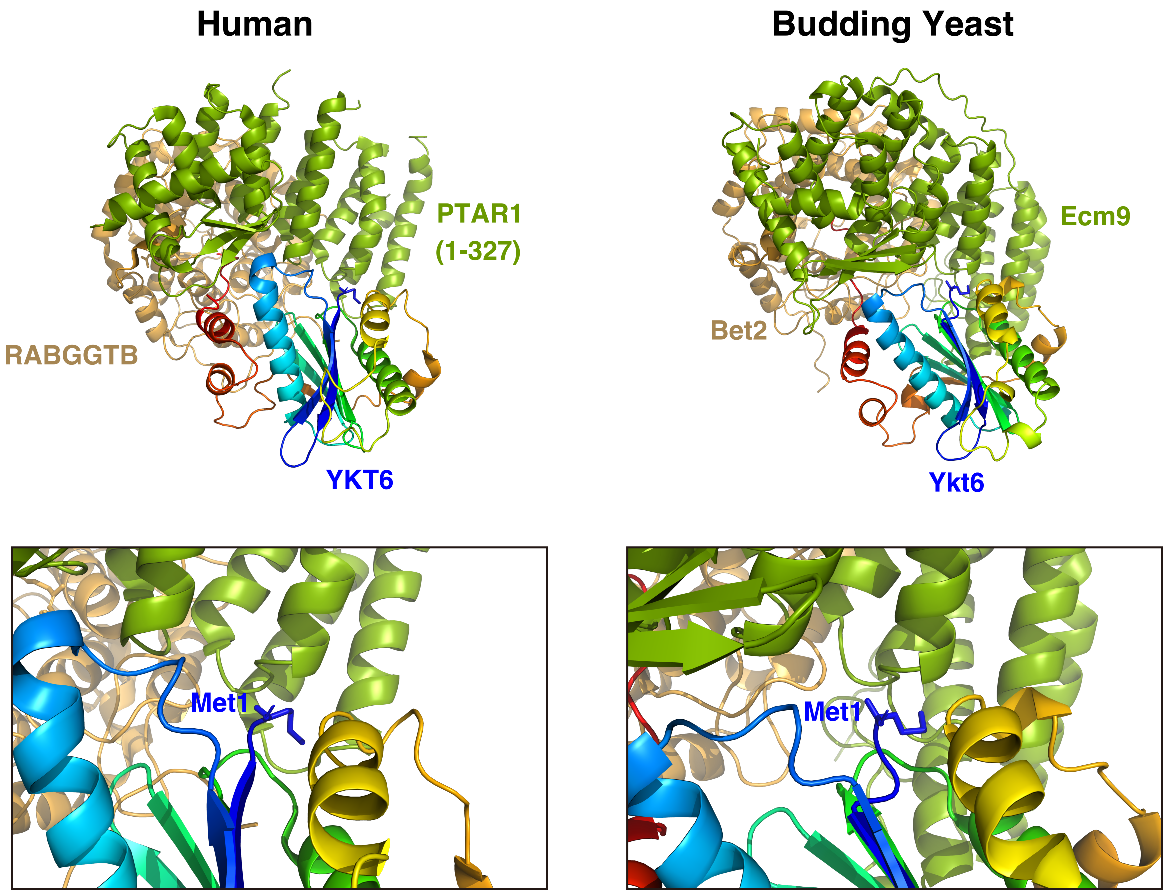

Figure S4. Structural comparison of the human and budding yeast GGT3–Ykt6 complexes. Structural comparison of the human GGT3–Ykt6 complex (PDB: 6J7F) and the budding yeast GGT3–Ykt6 complex (predicted by AlphaFold 3). PTAR1 and Ecm9 are shown in green, RABGGTB and Bet2 in orange, and Ykt6 in a rainbow color scheme. The lower panels are magnified views of the N-terminus of Ykt6. In budding yeast, the N-terminus of Ykt6 is located in close proximity of Ecm9, similar to its orientation in the human complex.
